# Results from Scotland's 2021 report card on physical activity and health for children and youth: Grades, secular trends, and socio-economic inequalities

**DOI:** 10.1016/j.jesf.2022.07.002

**Published:** 2022-07-19

**Authors:** Farid Bardid, Simone A. Tomaz, Avril Johnstone, Jenni Robertson, Leone C.A. Craig, John J. Reilly

**Affiliations:** a School of Education, University of Strathclyde, Glasgow, UK; b Faculty of Health Sciences and Sport, University of Stirling, Stirling, UK; c MRC/CSO Social and Public Health Sciences Unit, University of Glasgow, Glasgow, UK; d School of Pharmacy and Life Sciences, Robert Gordon University, Aberdeen, UK; e Institute of Applied Health Sciences, School of Medicine, Medical Sciences and Nutrition, University of Aberdeen, Aberdeen, UK; f Physical Activity for Health Group, School of Psychological Sciences and Health, University of Strathclyde, Glasgow, UK

**Keywords:** Physical activity, Sedentary behavior, Health, Childhood, Adolescence, Surveillance

## Abstract

**Background:**

The 2021 Active Healthy Kids Scotland Report Card aimed to identify secular trends and socio-economic inequalities, and to assess the physical activity and health of children and youth prior to COVID-19.

**Methods:**

An expert panel searched for data published in 2018–2020. Grades were assigned to nationally representative data using the Active Healthy Kids Global Alliance methodology.

**Results:**

The expert panel, following national consultation, awarded the following grades: Community/Environment B-, Organized Sport and Physical Activity B-, Government/Policy C-/C+, Active Transportation C-, Family/Peers D-, Recreational Screen Time F. Five indicators were graded inconclusive (INC): Overall Physical Activity; Active Play; Physical Fitness; Diet; Obesity. Grades have remained stable or declined, and surveillance has reduced, increasing the number of INC grades. There were marked socio-economic inequalities for eight indicators (Recreational Screen Time; Overall Physical Activity; Organized Sport & Physical Activity; Active Transportation; Diet; Obesity; Family/Peers; Community/Environment).

**Conclusions:**

Despite a decade of favorable policy, physical activity and health of children and youth has not improved, and marked socio-economic inequalities continue to persist in Scotland. There is a clear need for greater monitoring of physical activity and health, and improved policy implementation and evaluation, particularly as many indicators and related inequalities may have worsened following the COVID-19 pandemic.

## 1 Introduction

Levels of moderate-to vigorous-intensity physical activity (MVPA) are typically much lower than recommended, and levels of recreational screen time—used as a proxy for sedentary behavior (SB)—are well above recommended limits among children and adolescents globally.^1^, ^2^, ^3^ Previous Active Healthy Kids Scotland (AHKS) Report Cards^4^, ^5^, ^6^ found these low MVPA and high SB levels to be typical of Scottish children and adolescents too, despite favorable grades for influences on physical activity (PA) such as policy.^2^^,^^4^, ^5^, ^6^, ^7^ This may reflect limited policy implementation, sociocultural barriers, and lack of focus on some health behaviors (e.g., sedentary behavior) and age groups (i.e., under 5s).^8^^,^^9^

The period 2010–2020 was also characterized by significant changes in the PA and health landscape for children and adolescents in Scotland. For instance, UK government cuts in public spending and other policies have increased child poverty and health inequalities.^10^ Furthermore, screen-based devices for leisure have become more widely available.^11^^,^^12^ In contrast, favorable policy for PA, diet and obesity in Scotland might change PA and health positively by 2020 onwards.^13^^,^^14^ Additionally, the field of PA and health has evolved, with a better understanding of MVPA declines and SB increases across childhood,^15^^,^^16^ and growing evidence on the benefits of reducing screen time and increasing MVPA in children and adolescents.^17^, ^18^, ^19^

The present study aimed to report on the grades for PA and health indicators from the 2021 AHKS Report Card. Secondary aims were to (1) describe changes in report card grades over the decade prior to the COVID-19 pandemic, and (2) examine whether child and adolescent PA and health varied by socio-economic status (SES). This work is intended primarily to inform policy and practice, and support PA and health in Scotland, although the findings should have lessons for other nations. Previous AHKGA Global Matrix initiatives^2^^,^^7^^,^^20^ show that lessons can be learned from between-nation comparisons.

## 2 Methods

### 2.1 Procedure

The expert panel—representing different Scottish universities and expertise across different PA and health behaviors/outcomes—first convened in January 2021 to start developing the report card. The methods, developed by the Active Healthy Kids Global Alliance (AHKGA) and described previously,^2^ were followed.

Draft grades were assigned to each indicator by the end of May 2021. In June 2021, draft grades and rationale were disseminated widely to a range of stakeholder organizations and individuals from various academic fields, national and local governments, education, and non-governmental organizations with responsibility in sport, active transportation, and play. Full details of the assignment of draft grades and the consultation are provided on the project website (www.activehealthykidsscotland.co.uk).

An experienced member of the board of the AHKGA peer-reviewed the AHKS 2021 Report Card grades and process at the end of July 2021.

### 2.2 Data sources, benchmarks and grades

Scotland has several ongoing nationally representative surveys which are carried out for surveillance purposes. Three criteria were used to judge the suitability of data for grading. That is, data should be nationally representative, free of major biases/errors, and recent (i.e., data had to be available by May 2021 and collected between Autumn 2018 and prior to COVID-19 pandemic at the end of March 2020). For the *Policy and Government* indicator, the policies needed to be ‘active’ or current during this period. We summarized the data used to inform grades, but a full description of the selection and review process to inform the grades is given on the project website. Scottish Report Card grades are based on data from individuals aged 0–18 years.

Grading of indicators follows the AHKGA grading rubric, with grades corresponding to percentages of the population meeting an evidence-based benchmark (see Table 1).^2^ The Government and Policy Grade was based on the policy grading method by Ward et al.,^21^ which utilizes the Health-Enhancing Physical Activity Policy Audit Tool version 2^22^ and a scoring rubric. This involved the scoring of policy on different criteria: number and breadth of relevant policies, identified supporting actions, identified accountable organization(s), identifiable reporting structures, identified funding, and monitoring and evaluation plan. Weighting scores were assigned to each criterion, producing a total percentage score and corresponding grade.^21^

**Table 1:** Active Healthy Kids Global Alliance Grades and Interpretation.

Grade	Interpretation
	We are succeeding with a large majority of children:
A+	94%–100%
**A**	87%–93%
A-	80%–86%
	We are succeeding with well over half of children
B+	74%–79%
**B**	67%–73%
B-	60%–66%
	We are succeeding with about half of children
C+	54%–59%
**C**	47%–53%
C-	40%–46%
	We are succeeding with less than half of children
D+	34%–39%
**D**	27%–33%
D-	20%–26%
	We are succeeding with very few of children
**F**	<20%
**INC**	Incomplete Grade, where Scottish data were not available or were insufficient/inadequate to assign a grade

The AHKGA has 10 indicators^2^ listed in Table 2. The AHKGA *School* indicator in Scotland is included within the *Government and Policy* indicator as in previous report cards.^4^ Two additional indicators—diet and obesity—have also been included because of their importance to PA and health.^14^^,^^23^^,^^24^ This means that the AHKS 2021 Report Card comprises 11 indicators.

**Table 2:** Secular Trends in Active Healthy Kids Scotland Report Card Grades 2010-2020.

Indicator	**2013 Grade** *based on* *2010–2013 data*	**2016 Grade** *based on* *2014–2015 data*	**2018 Grade** *based on* *2016–2017 data*	**2021 Grade** *based on* *2018–2020 data*
Sedentary Behaviors	F	F	↑ D-	↓ F
Overall Physical Activity	F	F	F	INC
Organised Sport and Physical Activity	INC	INC	B	↘ B-
Active Play	INC	INC	D	INC
Active Transportation	C	C	C	↘ C-
Physical Fitness∗	—	—	INC	INC
Diet	D-	D-	↗ D	INC
Obesity	F	F	INC	INC
Family and Peers	D-	D-	↗ D	↘ D-
Community and Environment	B	B	↘ B-	B-
Government and Policy^†^	B	B	↓ C	C- Physical Activity C+ Diet and Obesity

∗The physical fitness indicator was not included in the 2013 and 2016 report card.

^†^The government and policy indicator did not include separate grades for physical activity and diet and obesity in the 2013, 2016 and 2018 report card.

For several indicators, data sources were informative—indicating secular trends in behaviors, or SES inequalities—even where an incomplete (INC) grade was assigned following the grading rubric. SES inequalities in some surveys were identified using the Scottish Index of Multiple Deprivation (SIMD), which considers income, employment, educational attainment, health, access to services, crime, and housing. The Health Behavior in School-aged Children survey (HBSC) Scotland^25^ uses an affluence scale to define SES, considering variables such as car ownership and vacations outside Scotland.

## 3 Results and discussion

### 3.1 Grades

***Sedentary Behavior*.** Only a single data source was identified—the HBSC 2018 Scotland report.^25^ The HBSC is limited to 11, 13, and 15 year olds and collected self-reported data on recreational screen time on weekdays only in three categories (TV, gaming, and other). Aggregated screen time data from the 2014 HBSC Scotland report suggests that recreational screen time on weekdays is around 9 ± 6 and 8 ± 5 h/day for boys and girls, respectively.^26^ As such, an F grade was assigned.

***Overall Physical Activity.*** The major national survey—the Scottish Health Survey (SHeS)—does not measure MVPA (‘no information on intensity of PA is collected’).^27^ Although the reported activities in the SHeS are assumed to be MVPA (as they are used to report on adherence to PA guidelines), it is more likely that most time in the activities reported will be of a light intensity, leading to a substantial overestimation of MVPA.^4^, ^5^, ^6^^,^^28^ Furthermore, the SHeS incorrectly applies the adult MVPA recommendation to 16–18 year olds (i.e., ≥150min of MVPA per week^29^^,^^30^), and child and adolescent MVPA recommendations to under 5s.^31^

The HBSC survey collects time spent in MVPA by self-report in 11, 13, and 15 year olds. Data from the 2018 HBSC Scotland Report indicates that only 17% of Scottish adolescents (19% boys and 15% girls) engage in at least 60 min of MVPA every day. These data have been used for grading of the MVPA indicator in the past, largely because the HBSC method has been validated for surveillance purposes.^4^, ^5^, ^6^ However, the HBSC uses a self-reported measure of achieving 60min of MVPA every day and the AHKGA benchmark now refers to the current PA guidelines of meeting an average of 60min of MVPA per day.^30^ In the absence of MVPA surveillance data from children, and the absence of adolescent data using the most recent PA guidelines/AHKGA benchmark, a grade of INC was assigned.

***Organized Sport and Physical Activity*.** Only one source—the SHeS 2019—measured participation in organized sport & PA; 66% of 2–15 year olds reported participation in any sport in the week prior to the survey).^27^ There was a lack of data for 16–18 year olds, though participation showed an age-related decline to age 15 years, so participation prevalence across the child-adolescent age range will probably be lower than 66%. Hence, a B- grade was assigned.

***Active Play*.** No suitable data were identified for the 2021 report card, justifying the INC grade. This indicator had been measured previously in surveillance and used in the AHKS Report Card 2018, but the surveillance of this indicator seems not to be continued in Scotland.

***Active Transportation*.** We found multiple suitable sources of surveillance data for active transportation to school. Three surveys (Hands Up Scotland 2019, HBSC 2018, and Scottish Household Survey [SHS] 2019)^25^^,^^32^^,^^33^ suggested that the usual mode of travel was active (on foot, by bicycle, or by scooter) in just over 40% of children and adolescents, justifying a C- grade.

***Physical Fitness*.** No nationally representative data on physical fitness in Scottish children and adolescents was found, and so an INC grade was assigned.

***Diet*.** The only suitable dietary surveillance data (i.e., SHeS) reported on fruit and vegetable consumption, which was much lower than recommended.^27^ In the absence of other dietary data, an INC grade was given.

***Obesity*.** We found data from 2 to 15 year olds from the SHeS^27^ as well as data from routine measurements of height and weight across the entire population in the first year of primary school.^34^^,^^35^ Data from SHeS 2019 suggests that 16% of 2–15 year olds (17% boys and 15% girls) have obesity as defined by BMI-for-age. However, the SHeS used BMI (instead of BMI-for-age) for 16–18 year olds and adopted incorrect reference data for under 5s. As such, the SHeS data for these age groups could not be used to estimate obesity prevalence. It should also be noted that BMI and BMI-for-age are conservative, with low-to-moderate sensitivity when identifying individuals with high body fatness.^36^ Obesity prevalence is therefore difficult to determine given these surveillance limitations; as such, an INC grade was assigned.

***Family and Peer Influence*.** Due to lack of parent-specific data, adult survey data were used. Adult health behaviors determine the environment in which children and adolescents grow up, and can influence their PA and health behaviors.^37^, ^38^, ^39^ The SHeS 2019^27^ found that 40% of men and 34% of women self-reported meeting the MVPA recommendation. Since the SHeS uses the adult PA recommendation from age 16 years upwards instead of the child and adolescent recommendation, these reported findings will include some adolescent data. Together with the use of self-report measurement, this will slightly overestimate compliance with the recommendations. The SHeS also found that adults self-reported a mean recreational screen time use of 5.4 h per weekend day and 6.2 h per weekday. Adult modelling of PA and screen time is therefore unfavorable. Only 22% of Scottish adults met the fruit and vegetable recommendation according to the SHeS 2019.^27^ The SHeS found that 29% of adults in Scotland have obesity according to their BMI, with two-thirds having overweight or obesity.^27^ The SHS 2019^40^ measured self-reported volunteering in sport and PA in adults: 5% and 2% of adult men and women reported having volunteered in sport or PA in the previous 12 months.

These data suggest that children and adolescents grow up in environments with low PA, high screen time, poor diet, and high body fatness among adults, and low MVPA and high screen time among peers. Moreover, current data suggests that these environmental factors are slightly worse compared to the previous report card, resulting in a D-grade.

***Community and Environment*.** This indicator refers to perceived safety, access to, and availability of outdoor/indoor spaces and opportunities for PA *in the local community*. In previous report cards, the SHS has provided a reasonable estimate of this indicator through parental reported perceived safety, access to, and availability of their children's play in their neighborhood. However, in recent versions of this survey these questions have been omitted. Since data from the 2018 Scottish report card was more comprehensive than the data available for the 2021 report card and this indicator tends to change slowly over time, previous data (SHS 2016^41^) was used along with newly available data resulting in a B- grade.

***Government and Policy*.** Scotland has many creditable national policies for PA. There appears to be many good links made between government directorates, policies, and organizations responsible for implementation. Additionally, there has been some level of implementation following Scotland's 2018 Physical Activity Delivery Plan.^8^ The launch of a new agency (Public Health Scotland) in 2020 is also notable, and further supports the Scottish Government's priority to have a more active and healthier Scotland. However, current policies provide limited information on how proposed actions will be monitored and evaluated in practice. As such, a C- grade was assigned for PA policy.

Relevant policies for diet and obesity have been implemented in Scotland. However, evidence of monitoring and evaluation and identified reporting structures is limited. Scotland's 2018 Diet and Healthy Weight Delivery Plan is a comprehensive document, and new policies/strategies in this area have emerged since the previous report card. This resulted in assigning a C+ grade for diet and obesity policy. The Good Food Nation (Scotland) Bill is currently being considered in Scottish Parliament.^42^ This places a duty on Scottish Government and other public authorities to produce national and local Good Food Nation Plans.^43^

### 3.2 Secular trends in grades

Table 2 shows grades for the indicators across all four Scottish report cards. There have been relatively few and only minor changes in grades with generally a slight decline in health behavior indicator grades. For *Sedentary Behaviors*, a secular trend was observed in HBSC Scotland with a slight increase in number of adolescent boys and girls exceeding 2 h per weekday watching TV from 2010 (66% and 64%, respectively) to 2018 (70% and 66%).^25^ Similarly, number of boys and girls exceeding 2 h per weekday playing computer games increased from 2010 (65% and 28%, respectively) to 2018 (71% and 42%).^25^ Regarding *Active Transportation*, the 2019 Hands Up Scotland Survey reported a slight decline in percentage of children using active modes of travel to school, particularly since 2014. Active travel has gone from 49.3% in 2010, through 50.4% in 2014, to 47.8% in 2019.^32^

### 3.3 Socio-economic inequalities

Table 3 reports on socio-economic inequalities for 8 indicators. There was no data available for *Active Play* and *Physical Fitness*; *Government and Policy* was also not covered*.*

**Table 3:** Socio-Economic Inequalities for indicators in the 2021 Active Healthy Kids Scotland Report Card.

Indicator	Benchmarks	Socio-economic status^a^	
		More deprived	Less deprived
Sedentary Behaviors	Exceeding 2 h/day of TV time	74%	60%
	Exceeding 2 h/day of gaming	61%	49%
	Exceeding 2 h/day of non-gaming computer time	67%	62%
Overall Physical Activity	Meeting physical activity guidelines	13%	19%
Organised Sport and Physical Activity	Not engaging in sport	53%	18%
Active Play		–	–
Active Transportation	Walking to school	63%	51%
	Cycling to school	1%	4%
Physical Fitness		–	–
Diet	Meeting 5-a-day fruit and vegetable recommendation	16%	17%
Obesity	Having obesity (children in primary 1)	14%	6%
Family and Peers	Adults volunteering in sport in past 12 months	16%	33%
	Going on outdoor excursions in past 12 months	4%	19%
Community and Environment	Living ≤5min from nearest greenspace	62%	67%
	Perceiving local area as safe	50%	72%
	Perceiving neighborhood as safe for children to play	75%	87%
	Perceiving outdoor space in neighborhood as good place to spend time	60%	73%
Government and Policy		–	–

a Number of children (or adults) meeting benchmarks.

For *Sedentary Behaviors*, SES differences for recreational screen time were observed in HBSC Scotland 2018,^25^ with the % of the sample exceeding 2 h/day on weekdays: 74% for TV in the lowest SES quintile vs 60% in the highest, 61% for gaming in lowest quintile vs 49% in highest, and 67% for other screen time in the lowest quintile vs 62% in the highest.

For *Overall Physical Activity*, HBSC Scotland also found that meeting the former MVPA guidelines (60min of MVPA per day) was lower in adolescents from the lowest SES quintile (14%) compared to the highest (19%).

For *Organized Sport and Physical Activity*, families in the lowest SES quintile reported lower engagement with organized sport and PA than those in higher SES in the SHeS, with 53% of children from the lowest quintile vs 18% from in the highest quintile.^27^

For *Active Transportation*, there are SES inequalities in active travel to school by cycling, with 1% of children from the lowest SES quintile cycling to school compared to 4% from the highest quintile. Importantly, access to bicycles varies greatly by household income: 62% of households with an income of >£50,000 have access to ≥1 bicycle, contrasted with 18% with an income of £10,000-£15,000.^33^ Interestingly, the SHS also reported that 63% of children from the lowest SES quintile walk to school compared to 51% from the highest quintile.^33^

For *Diet*, the SHeS reported a similar percentage met the 5 a day recommendation for fruit and vegetables in lowest (16%) and highest (17%) SES quintiles. However, those in the lowest SES quintile consumed slightly less fruit and vegetables (mean 2.8 portions/day in lowest vs 3.3 in highest SES quintile), and also had the most children consuming no fruit and vegetables in the last 24hrs (14% in the lowest vs 4% in highest SES quintile).^27^

*Obesity* prevalence varied by SES, with much higher rates among children of low SES. Obesity prevalence (i.e., BMI-for-age) in the first year of primary school was 13.3% in the lowest SES quintile vs 6.4% in the highest quintile.^34^ SES differences in child and adolescent obesity prevalence were also evident in the SHeS.^27^

*Family and Peers* influence also varied by SES. Prevalence of adult and child (peer) obesity is much higher in the lowest SES compared to highest SES groups. Dietary inequalities in Scottish adults by SES are also well established.^27^^,^^44^ Volunteering (including volunteering in sport) in the past 12 months is more prevalent among adults in the highest SIMD quintile (33%) compared to those in the lowest SIMD quintile in 2019 (16%).^40^ Outdoor excursions in the previous 12 months were reported much more commonly among the highest quintile for SIMD (19%) compared to the lowest SIMD quintile (4%).^40^

For *Community and Environment*, there was also evidence of SES inequalities. While most Scottish adults (66%) reported living within a 5min walk of their nearest greenspace in the SHS,^40^ this was slightly but significantly lower in the lowest SES quintile compared to the highest SES quintile (62 vs 67%). The HBSC 2018 survey^17^ reported marked differences between adolescents from the lowest and highest SES quintiles for overall perceived safety of their local area (perceived as generally safe by 50% vs 72%, respectively), whether they perceived their neighborhood as safe for children to play (75% vs 87%), and whether outdoor space in their neighborhood was perceived as a good place to spend time (60% vs 73%).

## 4 Conclusions

This fourth AHKS Report Card completes a decade of synthesis and critical appraisal of Scottish public health surveillance data for child and adolescent PA and health using the AHKGA methodology. Over the past decade, the key indicators have generally remained fairly stable or worsened slightly. Socio-economic inequalities are marked for many indicators. Surveillance of report card indicators remains limited or has worsened over the past decade. Some indicators have never been included in public health surveillance in Scotland (e.g., physical fitness), despite their importance to current and future health.^45^ It is also unclear whether current monitoring systems are set up to measure adherence to recent PA and SB guidelines for (young) children and adolescents.^29^, ^30^, ^31^ Importantly, there is limited data collected on under 5s, despite early childhood being recognized by WHO as “a period of rapid physical and cognitive development and a time during which a child's habits are formed”.^31^ Moreover, whilst there are creditable policies in Scotland with links to organizations responsible for implementation, few provide systems for monitoring and evaluation. Finally, lifestyle changes related to the COVID-19 pandemic since March 2020 may well have worsened many aspects of child and adolescent PA and health.^46^ In this regard, the present Report Card provides a basis to assess COVID-19 impacts.

Some recommendations can be provided to improve monitoring and to support decision-making in policy and practice in Scotland. First, national surveillance can be improved by revisiting existing surveys to capture and evaluate PA and health indicators more effectively (e.g., active play, SB, diet and obesity), and introducing new monitoring systems that assess other relevant PA indicators (e.g., physical fitness). Specific attention should be given to children under five years as there is limited data available on this age group.^31^ Second, existing national policies on child physical activity and health are broadly favorable although more focus is needed on policy implementation and evaluation. Recently, the 10.13039/100004423WHO has published a toolkit to assist policy makers and other stakeholders in implementing its guidelines on 10.13039/100006131PA, SB, sleep, and healthy eating in early childhood education and care settings.^47^ Third, practices in Scotland can be supported by reporting 10.13039/100006131PA and health indicators across age and sex, and monitoring and addressing health inequalities through national surveys. This will contribute to achieving the Sustainable Development Goals outlined by the United Nations and adopted by the Scottish Government.^48^^,^^49^

## Funding

FB, SAT, JR, LCAC, and JJR did not receive any funding for this work. AJ was supported by the UK Medical Research Council [MC_UU_00022/1; MC_UU_00022/4] and the Chief Scientist Office [SPHSU16; SPHSU19].

## Declaration of competing interest

The authors declare no conflict of interest.
